# Left Septal Fascicular Block: Myth Or Reality?

**Published:** 2003-07-01

**Authors:** Rex N MacAlpin

**Affiliations:** Professor (Emeritus) of Medicine/Cardiology, Division of Cardiology, Department of Medicine, David Geffen School of Medicine at UCLA, Los Angeles, California

**Keywords:** fascicular block, left septal fascicular block, intraventricular conduction defects, ventricular activation

## Abstract

Anatomic studies have shown that the left bundle branch divides into three fascicles in most humans. Changes in the 12 lead ECG (electrocardiogram) due to conduction abnormalities of the left anterior fascicle and left posterior fascicle are now part of the standard repertoire of electrocardiographic interpretation. There are no standard criteria for detecting conduction defects involving the third left fascicle, the septal or median fascicle, and the very existence of such defects is still a matter of controversy. The purposes of this article are to review the available evidence on this subject, suggest electrocardiographic criteria for its recognition, and present examples which illustrate that left septal fascicular block does indeed exist as a specific entity. Left septal fascicular block is a polymorphic conduction defect which may explain some previously inadequately understood electrocardiographic abnormalities.

## Introduction

**Figure 1:**
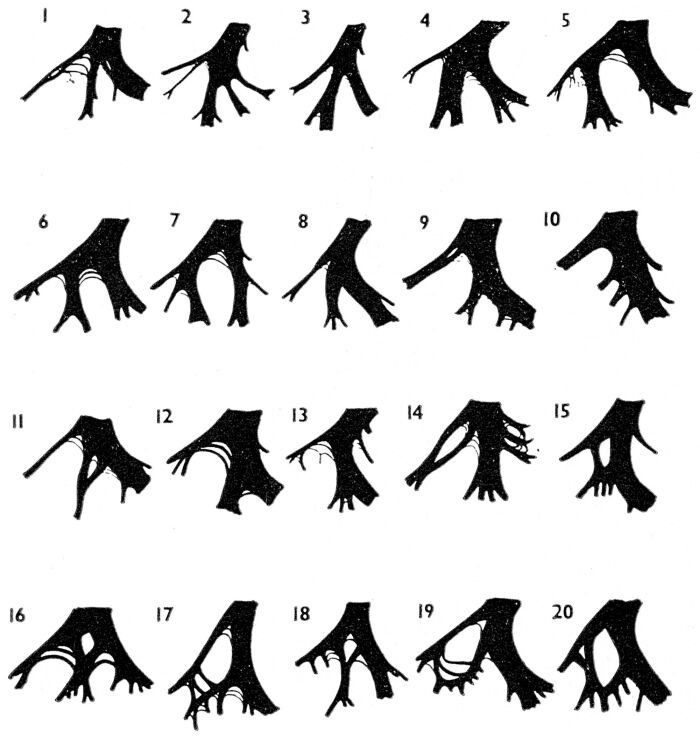
Diagrammatic sketches are shown of the left ventricular conduction system from 20 normal hearts. Sketches were derived by reconstructing the anatomy from serial histologic sections of carefully oriented blocks of left septal myocardium. Orientation is as if viewing the left septal surface from the left: anterior is to the viewer's left, posterior is to the right, and superior is at the top. In most of the examples shown, a middle or septal fascicle can be seen to arise from the central part of the main left bundle (sketches 1-4), from the anterior fascicle (sketches 5-7), from the posterior fascicle (sketches 8, 9, 11-14), or from fibers originating from both the latter fascicles (sketches 15-20). Reproduced from Demoulin JC and Kulbertus HE. Br Heart J 1972;34:807-14, by permission of the BMJ Publishing Group [12].

More than thirty years ago Rosenbaum, Elizari, and Lazzari [1,2] presented their monograph defining left anterior and left posterior fascicular blocks, or what they termed "the hemiblocks". These divisional blocks involving part of the left bundle branch system have become well-recognized aspects of clinical electrocardiography [3-6] Anatomic studies, however, have suggested that the termination of the left bundle branch itself is more complex than just a bifurcation [7-14]. Studies of the conduction system going as far back Tawara's work in 1906 [7] have indicated that as the left bundle branch divides, it gives off fibers to the left endocardial surface of the interventricular septum, as well as to its better recognized anterior and posterior divisions [7-14]. In some cases these septal fibers are distributed broadly over the left septal surface like an interconnecting network [7,8,11]. But the elegant histologic reconstructions of the left conduction system by Demoulin and Kulbertus [12] in 1972 showed that, although there is considerable variability in its anatomy, in most cases the septal fibers can be identified as a distinct third division of the left bundle branch, as shown in Figure 1.

In 1970 Durrer and his coworkers [15] described the activation sequence of the ventricles using isolated, perfused human hearts. They found that earliest activation of the left ventricle occurred, 0 to 5 ms after the onset of the left ventricular cavity potential simultaneously in three endocardial regions: an anterior paraseptal area just below the mitral valve, a posterior paraseptal area about one third of the way from apex to base, and the central region of the left septal surface. Right ventricular activation began about 5 ms later near the insertion of its anterior papillary muscle. It was concluded from these studies that these three areas of simultaneous left ventricular activation corresponded roughly to the insertions of the three fascicles that were the terminations of the left bundle branch [12,15]. It was also pointed out that the activation waves resulting from the anterior and posterior areas of initial left ventricular activation progress in directions approximately opposite to each other, and hence the resulting potential fields they create tend to cancel each other [9,16]. This leaves activation of the interventricular septum starting from its left septal surface to dominate the QRS in its first 10 ms, despite a small, opposite, slightly delayed potential from activation of the right septal surface via the right bundle branch [3,15]. This produces initial QRS forces directed to the right and anteriorly, causing initial negative deflections in left-to-right oritented leads, q waves in leads I, V5-V6. These q waves have been termed "septal q waves" [17]. This model of ventricular activation incorporates a quadrafascicular picture of the ramifications of the His bundle: right bundle branch and three fascicles of the left bundle branch [15,18]. It is helpful to have this trifascicular pattern of the left bundle branch divisions in mind when trying to understand the changes that occur in direction of initial QRS forces with left anterior and left posterior fascicular blocks [2-5,9,16,19]. The term "hemiblock" is therefore no longer felt appropriate in describing these left intraventricular conduction abnormalities [12,20].

The QRS alterations in left anterior and left posterior fascicular blocks are well described not only in the monograph of Rosenbaum, Elizari, and Lazzari [2], but also in medical articles [19], and texts of electrocardiography and vectorcardiography [3-6,16]. There are reasons why such agreed upon criteria do not exist for block of the third division of the left bundle branch, left septal fascicular block (LSFB). The anatomy of this fascicle, where it exists as a distinct entity, varies more than that of the other two left fascicles [12]. These septal fibers usually have many interconnections [8,12]. This makes some persons less likely than others to suffer block of this division of the left bundle branch. Septal fibers of the left bundle branch may have the shortest refractory period of conduction fibers originating from this bundle branch [21,22]. Various criteria for LSFB have been proposed based on case reports, [11,23-29] on consequences of experimental incision of septal fibers in the dog, [30,31] on electrical-anatomical models, [9] and on computer-based projections of QRS morphology [32,33]. It may present with multiple morphologies, and is frequently combined with other conduction abnormalities which obscure its presence [9,23,34]. Popular textbooks of electrocardiography tend to ignore the possibility of its existence [5], to dismiss it as unproven [6], controversial [4], or to maintain it cannot be diagnosed [4,35]. When it is mentioned, the coverage is brief [3,6,36].

One major reason for the multiple morphologies with which LSFB may present is that septal activation is normally accomplished by a double envelopment starting from both left and right septal surfaces as contrasted to the single, endocardial to epicardial direction of activation of the free walls of the two ventricles [37-39]. Reference to a diagrammatic depiction of normal ventricular activation in 2A illustrates this point. Activation of the right septal surface is initiated by septal branches of the right bundle branch, starting no more than 5 ms after first activation of the left septal surface [15,37]. The activation wave traverses the septum in either direction in about 40 ms, so that septal activation is nearly completed within 35 ms of the beginning of ventricular depolarization [15,37], except perhaps for parts of the basal septum, which has a relative dearth of Purkinje fibers leading into it [3,11,40]. By the time instantaneous QRS vectors are dominated by left ventricular free wall activation and have already rotated to the left at 40 ms, septal activation has been almost completed and will have little effect on the direction or magnitude of terminal QRS forces [37].

The amount of the septum normally activated via the right bundle branch varies between individuals [15]. Orientation of the septum also varies, facing to the right, anteriorly and inferiorly in horizontal hearts, and anteriorly, superiorly, and either to the left or right in vertical hearts [41]. As the direction of initial QRS forces is related to the orientation of the septum [42], the effect of LSFB on the ECG may vary with septal orientation.

## Possible Models of LSFB

**Figure 2:**
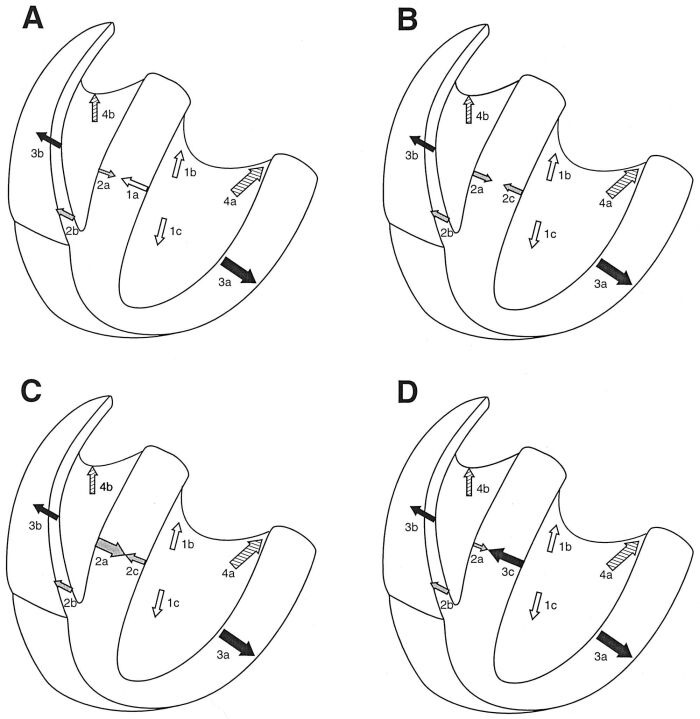
**A**. Schema of normal ventricular activation. Vectors 1a, 1b, and 1c (clear arrows) are forces produced by initial depolarization of the left ventricle via left septal, left anterior, and left posterior fascicles respectively during the first 10 ms of the QRS. Vectors 2a and 2b (dotted arrows) represent forces generated respectively by right septal and right ventricular apical and free wall activation via the right bundle branch starting no more than 5 ms after initiation of left ventricular activation. Vectors 3a and 3b (solid black arrows) represent forces generated during the middle of the QRS by activation respectively of the left and of the right ventricular free walls. Vectors 4a and 4b (arrows striped diagonally) represent activation respectively of the posterobasal portions of the left ventricle and outflow tract of the right ventricle during inscription of the terminal part of the QRS. **B**. Slight delay in left septal activation (mild LSFB): forces produced by diminished left-to-right and augmented right-to-left septal activations balance each other, with resulting loss of septal q waves. **C**. A greater delay in left septal activation can result in predominantly right-to-left and front-to-back septal depolarization, which may produce q waves in right precordial leads, in addition to loss of septal q waves. **D**. When for some reason right septal activation remains limited in scope or is delayed, loss of initial left septal activation from LSFB results in late left-to-right and back-to-front septal activation. The latter derives from activation waves moving into the septum from myocardium activated initially via left anterior and posterior fascicles. This deviates the mid-late QRS forces anteriorly. Septal q waves should be missing, and right precordial q waves may or may not be present.

Based on this model of ventricular activation, Figure 2 illustrates a range of possibilities with LSFB. In Figure 2A is represented the normal sequence of ventricular activation. In Figure 2B, the delay in left septal activation is only slight, such that the potentials arising from the activation wave proceeding from left-to-right are exactly balanced by those generated by the right-to-left activation wave. This would result in a leftward shift of initial QRS forces, with abolition septal q waves, but with little other change in QRS configuration or duration [9]. In Figure 2C the delay in left septal activation is greater, resulting in dominant right-to-left and front-to-back septal activation, yielding initial QRS forces directed to the left and posterior. If not counterbalanced by simultaneous anteriorly directed forces from right ventricular activation, this would result in q waves in right precordial leads, particularly V1 and V2, in addition to the absence of septal q waves from left oriented leads, and possibly to increase in magnitude of leftward and posteriorly oriented QRS forces (increased depth of S in right precordial leads) at 30 to 40 ms from the start of ventricular activation. In Figure 2D is depicted what might be expected when left septal activation is blocked in a situation where for some reason right-to-left septal activation is attenuated or delayed (e.g., with some degree of right bundle branch block). In this case septal activation is significantly delayed and has to be initiated from depolarization waves reaching it from left ventricular myocardium which has been previously activated by anterior and posterior fascicles of the left bundle branch. Initial QRS forces will likely be directed leftward, with disappearance of septal q waves. Left-to-right and posterior-to-anterior septal activation will occur later than left ventricular free wall activation, and will be relatively unopposed by any right-to-left septal activation. Depending on the degree of this delay, this may produce only a decrease in depth of right precordial S waves, or might actually result in an anterior shift in mid-to-late QRS forces with an increase in their anterior magnitude, somewhat analogous to the left-superior and right-inferior mid-to-late QRS shifts seen respectively with left anterior and left posterior fascicular blocks. Unfortunately, in some cases this would be masked by the coexisting right bundle branch block (RBBB). Such prominent anterior forces, otherwise unexplained by right ventricular hypertrophy or posterior infarction, have been claimed in anecdotal cases to be the result of just such a LSFB [11,12,23-27]. That some of these were shown to be associated with RBBB when the conduction defect worsened is consistent with this hypothetical model [25,26]. A transient anterior shift of QRS forces has been seen during myocardial ischemia produced by occlusion of the anterior descending artery during balloon angioplasty, possibly due to block of right septal activation [43]. Other studies have shown that otherwise unexplained prominent anterior forces can be a normal variant without unfavorable clinical implications [44].

## Proposed Criteria for LSFB

In Table 1 are listed proposed criteria for diagnosis of LSFB based on the above considerations. The major criterion is based on changes in the first part of the QRS: septal q waves are markedly diminished or lost by shift in initial QRS forces to the left, and in some cases also posteriorly due to loss or frank reversal of initial left-to-right septal activation [9,29,32,34,46]. This means loss of q waves from I, V5-6, and/or from inferior leads in cases with vertical heart orientation. Q waves may develop in V1-2 especially when anteriorly directed forces of right ventricular free wall activation fail to overbalance the posteriorly directed forces of "right-to-left" septal activation. With intact right bundle branch conduction, this may produce qrS complexes in V1-2, but QS complexes can occur, which mimic the chronic picture of anterior infarction. It must also be differentiated from complete or incomplete LBBB, ventricular pre-excitation, congenitally corrected transposition of the great vessels, effects of left or right ventricular hypertrophy, and orthotopic cardiac transplantation among other conditions. As right septal activation still starts via the right bundle branch no more than 5 ms after the start of left ventricular activation, a moderate delay in activation of its left septal surface would not be expected to increase the QRS duration importantly. Mean frontal plane QRS axis and ventricular activation times in aVL, aVF, and V5-6 should remain normal, as activation of the left ventricular free wall and apex via the left anterior and posterior fascicles is undisturbed. Absence of slurring or delay in R wave upstroke in leads overlying the left ventricle helps to differentiate this conduction defect from various degrees of incomplete LBBB [46]. Prominent anterior shift in mean QRS vector, manifested as prominent R waves with increased R:S ratio in V1-2, can occur when right septal activation is blocked or otherwise limited, resulting in relatively late, predominantly left-to-right and posterior-to-anterior septal activation. This is more likely to be seen when there is coexistent delay of conduction in the right bundle branch.

## Illustrative Examples Of Probable And Possible LSFB

### Absent Septal Q Waves in Otherwise Normal ECGs: LSFB, Pure Septal Infarction, or a Normal Variant?

Absence of septal q waves in ECGs that are otherwise normal is the simplest way that LSFB might present. In prior work, comparison was made of 92 consecutive cases showing this pattern with age- and gender-matched "controls" with normal tracings including the presence of septal q waves [47]. No significant difference was found in incidence of overt cardiac disease. Moreover, this did not seem to be an acquired ECG pattern, as the age distribution in cases with normal ECGs except for absence of septal q waves was identical to that of a larger group with normal ECGs including presence of septal q waves. Thus the absence of septal q waves in ECG's that are otherwise completely normal in most instances probably represents a variant of normal found in about 7 percent of normal electrocardiograms. This is consistent with vectorcardiographic studies in normal populations [48-50]. However, the identical ECG pattern might result from myocardial infarction limited to the interventricular septum [51]. In this case, comparison with prior tracings and clinical correlation might be needed for certain differentiation.

### LSFB without Other Fascicular Conduction Defects

**Figure 3:**
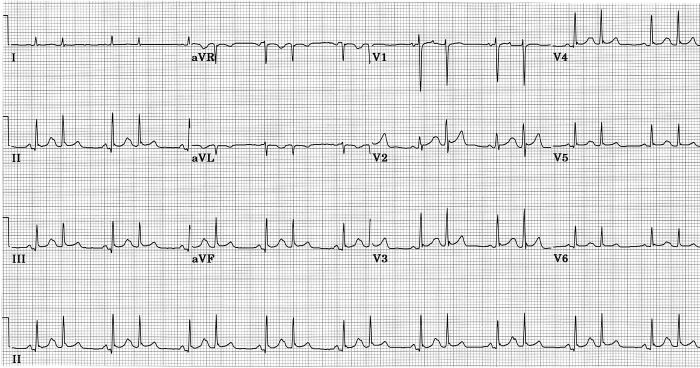
Aberrant ventricular conduction following premature atrial beats causes loss of septal q waves without major change in mean frontal plane QRS axis, probably due to transient LSFB [45].

However, such a pattern can result from an aberration in intraventricular conduction [52]. In Figure 3 such an example is shown in a 20 year old woman suffering an adult respiratory distress syndrome following her second stem cell transplant for recurrent acute myelogenous leukemia. She had no signs or symptoms of cardiovascular disease. In this case premature atrial beats were conducted with slight aberrancy, the major result of which was disappearance of q wave from leads II, III, aVF, and V4-V6, leaving septal q waves absent. QRS duration and frontal plane mean QRS axis were unchanged. There was some increase in amplitude of anterior QRS forces at about 30 ms. The aberrant conduction was probably due to LSFB, with that fascicle in this case having the longest refractory period of the four fascicles. Aberrant beats remained otherwise within normal limits, without secondary ST or T changes. Twelve minutes after this ECG she developed atrial fibrillation with a ventricular rate of 195 per minute; at that time all beats showed the same aberrant conduction as was seen only on premature beats in the preceding ECG. If such an aberrant form of intraventricular conduction were to become permanent, without other abnormalities, and without prior tracings, there would be no way to separate the resulting ECG from a normal variant as described in the preceding paragraph, or from the result of a pure septal infarction [51-56].

**Figure 4:**
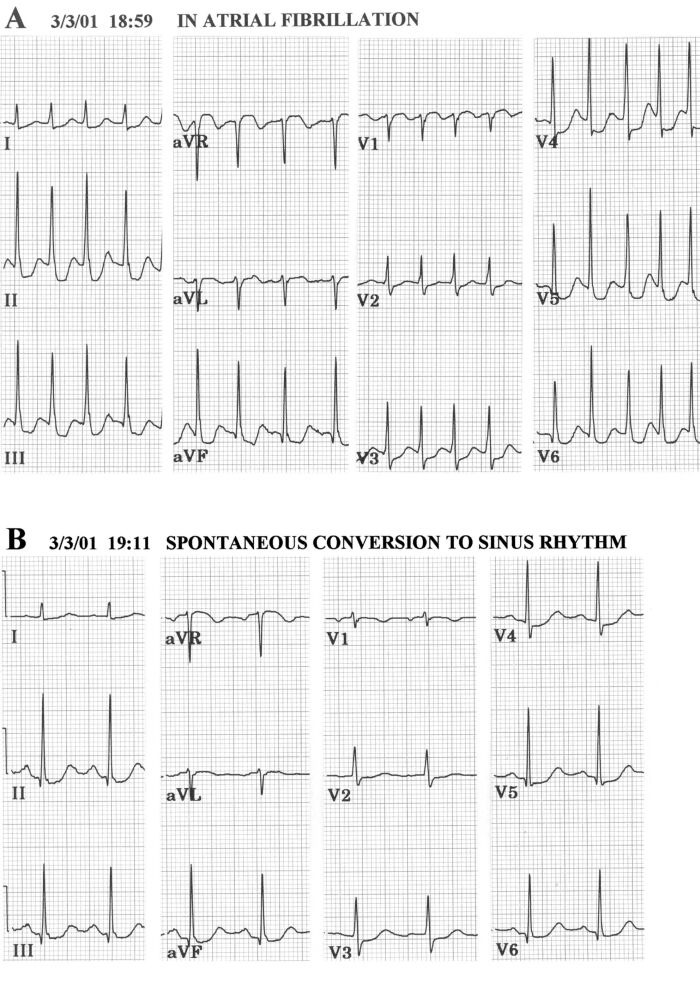
**A**. Variable aberrancy of intraventricular conduction during rapid atrial fibrillation produces loss of septal q waves from leads II, III, aVF, V5 and V 6, with decrease in r in V1 but no change in QRS axis in beats with short R-R intervals. An example of intermittent LSFB. **B**. Following spontaneous conversion to sinus rhythm 12 minutes after ECG shown in A., rate slows and prominent septal q waves are present in II, III, aVF, V5 and V6 [45].

In my experience, it was not hard to find examples of this type of transient LSFB [52]. It was seen most commonly in subjects with atrial fibrillation, particularly with rapid ventricular rates. Short R-R intervals would result in disappearance of septal q waves, as shown in the example of Figure 4. This was taken on a 69 year old woman who experienced paroxysmal atrial fibrillation without any prior cardiac history. Presence of prominent septal q waves in inferior leads and V5-V6 was seen in the second ECG taken 12 minutes later, after spontaneous conversion to sinus rhythm. An echocardiogram was normal except for presence of left atrial enlargement. Response of left ventricle to dobutamine stress was normal. Rate-related, transient loss of septal q waves in every beat was also encountered in some cases of supraventricular tachycardia or atrial flutter with 2:1 atrioventricular block [52]. 

**Figure 5:**
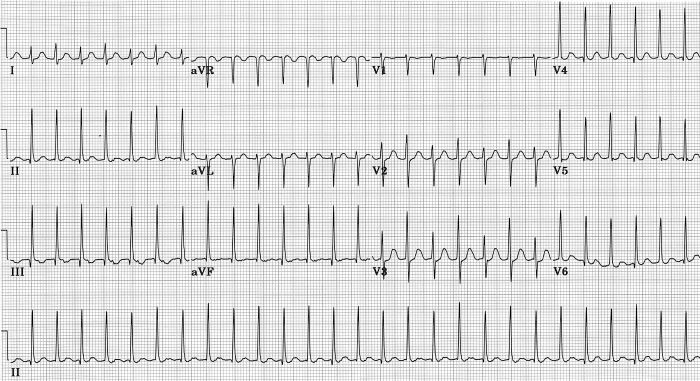
During an episode of AV nodal reentrant tachycardia, absence of septal q waves and anterior shift of early-mid QRS forces are seen in every other beat; possible rate-related 2:1 LSFB.

Figure 5 shows an ECG during supraventricular tachycardia in a 27 year old woman complaining of recurrent palpitations. In every other beat, septal q waves are absent along with slight increase in anteriorly directed forces. This is a rate-related 2:1 LSFB, a form of electrical alternans. Following conversion to sinus rhythm, all beats had septal q waves. She had no clinical or echocardiographic evidence of structural heart disease. Subsequent electrophysiological study showed this to be an atrioventricular nodal re-entrant tachycardia of the usual variety. Attacks were abolished with radiofrequency catheter AV nodal modification.

**Figure 6:**
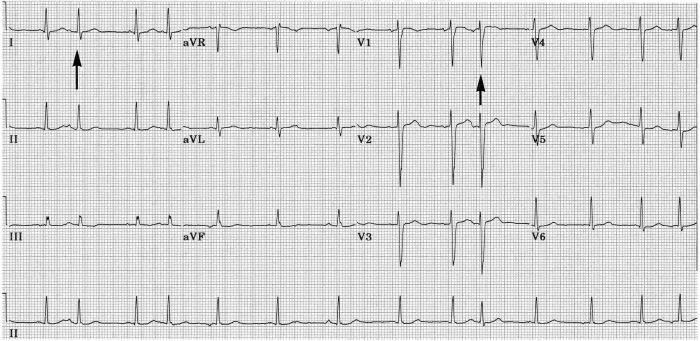
Ectopic atrial beats with varying degrees of prematurity causing varying aberrancy with slight variations in the initial and middle parts of the QRS without major changes in mean QRS axis. At the first vertical arrow, septal q waves disappears for a beat. At the second vertical arrow, at shorter preceding R-R interval, a small q wave appears in V1 - V3, and S wave in V3 is deeper, indicating possibly a greater degree of LSFB [45].

In other cases the change in initial QRS direction in aberrantly conducted beats can vary with degree of prematurity of the ectopic atrial beats. Figure 6 is an electrocardiogram from an 75 year old man with severe disease of his peripheral, carotid and coronary arteries. His echocardiogram showed a left ventricle with inferior and posterobasal hypokinesis, and an ejection fraction of 45%. Some aberrantly conducted beats lacked septal q waves. In others septal q waves were markedly attenuated. With the most premature ectopic atrial beat which occurred while right precordial leads were being recorded, an initial q appeared in leads V1-V3, suggesting posterior shift of initial QRS forces, which was probably due to a greater degree of LSFB.

**Figure 7:**
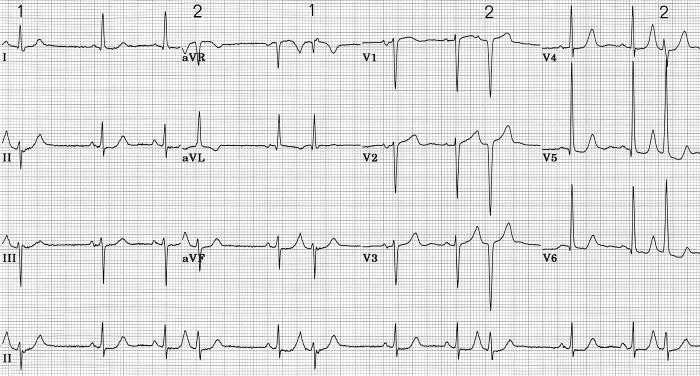
Two types of aberrant intraventricular conduction are caused by premature atrial beats. There is underlying left ventricular hypertrophy. In beats labeled 1, there is no change in direction of initial QRS forces, but development of left and superior mean QRS axis and prolongation of the QRS indicate transient LAFB accompanied by RBBB. In beats labeled 2, initial forces shift leftward and posteriorly without change in mean QRS axis, and without measurable prolongation of QRS. Loss of septal q and of initial r in V1-V3 mimics anterior infarction, but is probably due to transient LSFB [45].

The example shown in Figure 7 was from a 90 year old woman with a past history of hypertension, paroxysmal atrial fibrillation, and Parkinsonism,. Her echocardiogram showed left atrial enlargement, but left ventricular size and wall motion were normal despite the presence of electrocardiographic voltage criteria for left ventricular hypertrophy (LVH). Atrial premature beats cause two distinct forms of aberrant QRS complexes. The first type was not associated with discernable change in direction of initial QRS forces, but was associated with development of marked left and superior axis deviation and new broad terminal S in I and slurred terminal R in aVR consistent with left anterior fascicular block (LAFB) combined with RBBB. The second type, in which the ectopic atrial beat occurred slightly earlier, was associated with loss of septal q waves from aVL, V5 and V6, loss of r waves in V1-V3, but no major change in frontal plane mean QRS axis or QRS duration. There was increase in depth of S waves in V1-V4, and in height of R waves in V5 and V6. It would be natural to claim that the latter type of aberration in Figure 7 was due to incomplete LBBB, but such is not likely in that the neither ventricular activation time nor total QRS duration was changed, nor was there slurring of R wave upstroke in V5 or V6 in aberrantly conducted beats. This is more likely another example of LSFB. The resulting QRS configuration mimics an anterior infarct, and augments the voltage signs of LVH. Diminution in amplitude or complete loss of septal q waves in the systolic overload pattern of LVH [57] has been ascribed to augmented left ventricular free wall forces that are not counterbalanced by left-to-right septal forces [58]. It is more likely that the loss of septal q waves in this situation is the result of a conduction defect, LSFB [5,29]. This type of aberrancy has been produced with programmed premature electrical stimulation of the right atrium in subjects with known LVH [29], and has been reported after spontaneous early premature atrial beats [28]. This manifestation of LSFB may be a major cause of QS complexes that can occur in V1-V2 in some cases of severe LVH [3,29,59,60]. It could also contribute to the low specificity of QS complexes in V1-V2 and of absent septal q waves for diagnosing septal or anterior infarction [51,59-63].

**Figure 8:**
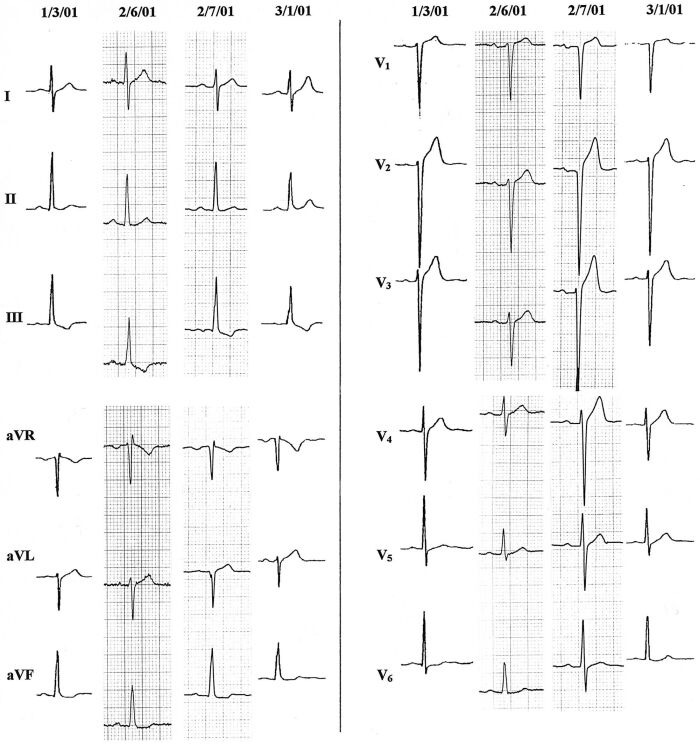
Loss of initial r in V1 - V2 and of septal q waves result from balloon aortic valvuloplasty on 2/6/01 in a 17 year old boy with severe aortic stenosis and left ventricular hypertrophy. Limb leads of 4 sequential tracings are on the left half of the figure, and precordial leads are on the right. Resulting QRS on 2/7/01 resembles pattern of anteroseptal infarction, but was most likely due to LSFB, with partial recovery by the time of the last ECG [45].

The series of ECGs shown in Figure 8 probably also demonstrate this type of LSFB complicating aortic balloon valvuloplasty in a 17 year old boy with severe congenital aortic stenosis. Two preoperative tracings showed LVH with tiny q waves in leads I and V6, and initial r waves in V1-V2. Following the valvuloplasty procedure on 2/6/01, he lost his initial r waves in V1-V2, and q waves were no longer seen in I and V6 (although q waves persisted in inferior leads). QRS duration and ventricular activation time in V5 and V6 did not change. Postoperative troponin I levels peaked at 0.54 ng/ml (normal ≤ 0.1) with normal total creatine kinase (CK) and CK-MB values, so anterior or septal infarction was unlikely to have been the cause of the ECG change. No ST-T changes evolved. The fourth tracing in the series, taken 3 weeks later, showed return of initial r wave in aVL and V2, but QS remained in V1, and clear septal q waves had not returned to V6.

### Combination of Left Septal Fascicular Block with Other Left Fascicular Blocks

The presence of four fascicles (three left and one right) rather than the traditional three, adds complexity to the possible combinations of fascicular blocks [9] In Table 2 are listed the resulting 15 possibilities for mono- and poly-fascicular blocks. This listing would be even more complex if fascicular blocks were further subdivided into incomplete and complete varieties.

**Figure 9:**
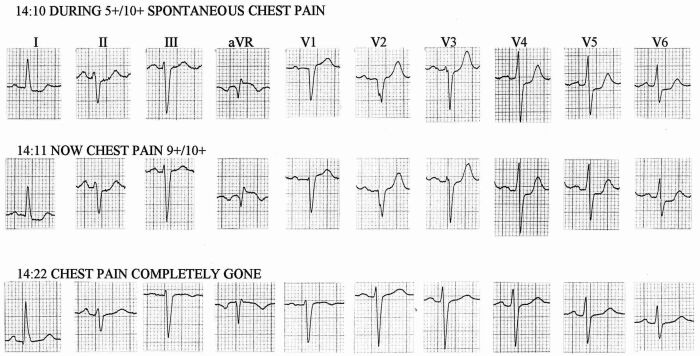
Three sequential ECGs during a spontaneous anginal attack in a patient with a baseline chronic LAFB. Electrode positions were unchanged during the 12 minutes covered by these tracings. Although other explanations are possible, transient loss of septal q from lead I and of initial r from V1 - V2 suggest transient LSFB caused by ischemia superimposed on fixed LAFB, briefly mimicking anterior infarction [45].

Figure 9 shows three sequential ECGs taken on a patient during a bout of spontaneous angina. This 60 year old woman had mixed angina with a 50 percent diameter stenosis of her proximal anterior descending coronary artery, and an 80 percent ostial right coronary arterial stenosis. Her baseline ECG showed LAFB. On the day of the tracings in Figure 9 an episode of spontaneous angina occurred in her cardiologist's office. The three ECGs shown were recorded with the patient supine on an examining table, without moving the electrodes between tracings. As her angina was waxing, her ECG showed LAFB without q waves in lead I, with QS deflections in V1-V2, and ST segment depression that was downslanting in I, nearly horizontal in V6, and upsloping in V4-V5. After recovery from the attack, shown in the third tracing, a q wave in lead I, and initial r waves in V1-V2 had reappeared, and ST depression had disappeared, leaving her ECG exactly as it had been on contemporary but prior tracings. In retrospect, the transient ECG changes had also been associated with decrease in S wave amplitude in V2-V3. The initial ECG of this series looks like LAFB complicated by anterior infarction with lateral subendocardial ischemia. But the transient nature of the QS in right precordial leads and loss of q in lead I could be due to transient LSFB, a conclusion suggested by others describing similar cases [64,65]. One could also argue convincingly in this setting that the QRS changes were due to left septal "parietal" block, given the presence of myocardial ischemia at the time [33], or to severe ischemia causing septal muscle to be transiently electrically inert [28,66-68]. If the changes seen were due to LSFB, this case would illustrate that superimposed on a chronic LAFB, a transient LSFB can be associated with a leftward and sometimes also posterior rotation of initial QRS forces, as would be predicted from the hypothetical model described above. This raises the possibility that a leftward orientation of initial QRS forces in LAFB, deemed inconsistent with a diagnosis of LAFB by Rosenbaum et al. [69], but seen in a large fraction of cases of this conduction abnormality [70-73], could in some cases be due to LAFB combined with involvement of left septal fibers, as suggested by others [32,71], particularly when q waves are present in right precordial leads. By reference to Figure 1, it is easily seen how a discrete lesion in the region of the left anterior fascicle could produce such a conduction defect, when the bulk of septal fibers of the left bundle branch originate as a branch of the left anterior fascicle.

**Figure 10:**
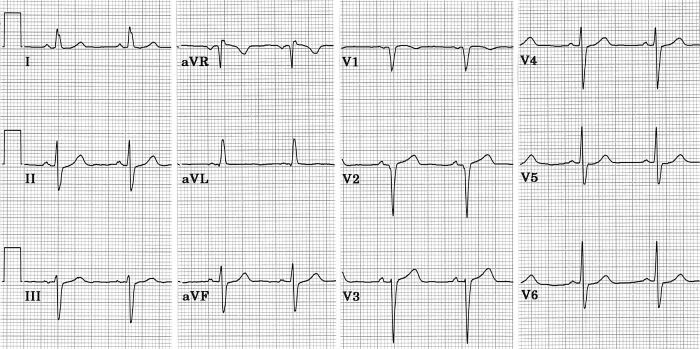
Pattern which may be due to chronic and fixed LSFB combined with LAFB in a patient with aortic stenosis, left ventricular hypertrophy, but normal coronary arteries and left ventricular wall motion. Pattern mimics anteroseptal infarction with LAFB.

Figure 10 illustrates a case with a chronic pattern consistent with LAFB combined with LSFB in an 83 year old man with severe aortic stenosis, concentric left ventricular hypertrophy, but normal coronary arteries and left ventricular wall motion. Initial QRS forces were directed to the left, inferiorly and possibly posteriorly. QS deflections in V1-V2 mimicked anterior infarction, although there was no clinical or echocardiographic evidence of prior myocardial infarction.

I have yet to find examples that could convincingly be called left posterior fascicular block (LPFB) combined with LSFB, although such probably exist. Part of the problem is that septal q waves in left facing leads most commonly diminish or disappear when isolated LPFB develops, due to superior and usually leftward shift of initial QRS forces [2,9,16]. This means the addition of LSFB to existing LPFB might not cause any recognizable change in QRS configuration. One can speculate, however, that when LPFB is present, in the absence of intervening infarction, the diminution or disappearance of q waves from inferior leads and of r waves from right precordial leads might indicate the addition of LSFB.

### LSFB Combined with RBBB with or without Other Left Fascicular Blocks

The presence of RBBB is a special case in the detection of coexisting LSFB. RBBB alone may cause slight decrease in amplitude of initial r waves in V1-2, but does not change the direction of initial QRS forces [5,29,46,74-76]. Hence, septal q waves are preserved when RBBB occurs [46,74,76]. In the special case of RBBB combined with LSFB, both right ventricular and interventricular septal activation are dependent on delayed left-to-right and back-to-front septal activation via depolarization waves originating from myocardium activated originally through the left anterior and posterior fascicles. Assuming a block of the proximal right bundle branch, there will be no early right-to-left component to septal activation, which must thus occur asynchronously later than the start of depolarization of the free wall of the left ventricle, and is a necessary prelude to depolarization of the right ventricular free wall. Direction of initial QRS forces will depend on the orientation of net potentials generated by the start of left ventricular activation via the left anterior and left posterior fascicles. This might well vary from person to person, but will not be modified by early forces from right ventricular free wall activation. It probably would be directed to the left [9], but conceivably could point either anteriorly or posteriorly, superiorly or inferiorly. Thus septal q waves would likely be absent from left facing leads, which is not an expected effect of uncomplicated RBBB [46,74-76]. But the presence or absence of initial r waves in right precordial leads would be hard to predict. Their absence when RBBB exists would add weight to a presumptive diagnosis of coexistent LSFB, but their presence would not necessarily rule it out.

The direction of forces in the middle of the QRS in this setting would be deviated anteriorly by delayed and relatively unopposed left-to-right and back-to-front activation of the interventricular septum. But this would be hard to separate from the anterior and rightward terminal QRS forces due to delayed activation of the right ventricular free wall. Hence, RBBB combined with LSFB might look just like RBBB except for atypical alterations in the direction of initial QRS forces.

**Figure 11:**
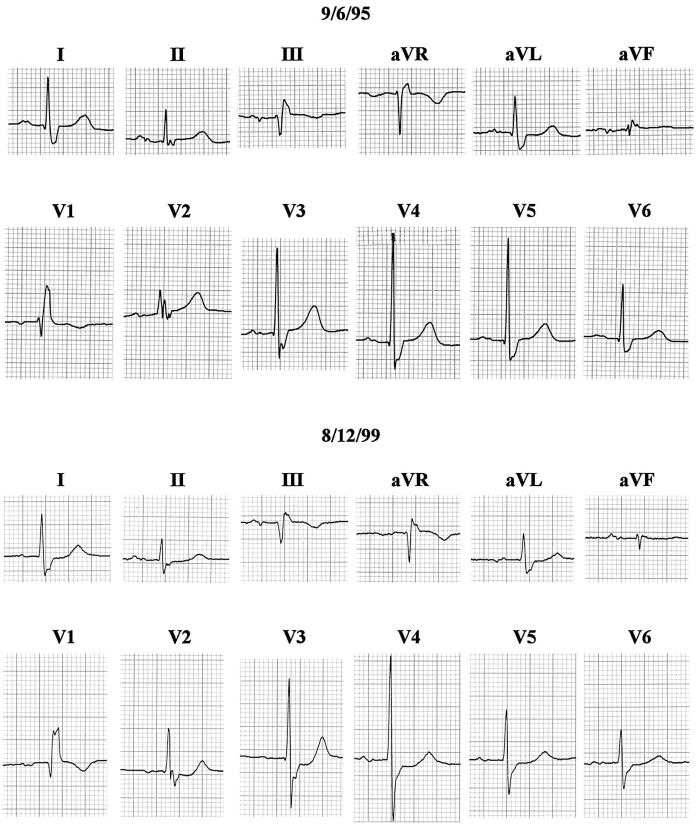
Tracings before (top) and after (bottom) development of LSFB in a woman with hypertrophic cardiomyopathy and a fixed pattern of RBBB. LSFB resulted in loss of septal q from I and V6, and loss of initial r in V1 - V2. Left ventricular wall motion remained normal, and there was no clinical evidence of intervening infarction. See text for details [45].

Figure 11 shows ECGs from a 96 year old woman with asthma, hypertrophic cardiomyopathy, and moderate aortic stenosis. Diastolic thicknesses of interventricular septum and posterior left ventricular free wall in 1999 were 13 mm and 12 mm respectively. Systolic anterior motion of the mitral valve was seen intermittently and was associated with a moderate systolic pressure gradient in the aortic outflow tract. Left ventricular wall motion and ejection fraction were normal. She never had hypertension or a clinical myocardial infarction. The two tracings shown are examples of the numerous ECGs she had at two periods of her life. The first one dated in 1995 is typical of her ECGs prior to 1998. It showed high voltage of left ventricular hypertrophy and a typical RBBB. Initial QRS forces were directed to the right, anteriorly and slightly superiorly, resulting in clear septal q waves in left facing leads, and initial r waves in V1 and V2. All ECGs after 1998 were like the second one shown in figure 11 dated in 1999. Initial QRS forces were directed to the left, probably slightly posteriorly, but still slightly superiorly. Septal q waves had disappeared from left facing leads, and in right precordial leads initial r waves had been replaced by q waves. S waves in the mid-precordial leads were deeper. There were no significant changes in her echocardiograms between these two tracings. The change in direction of initial QRS forces responsible for these ECG changes is almost certainly due to the superimposition of LSFB on the RBBB [9].

**Figure 12:**
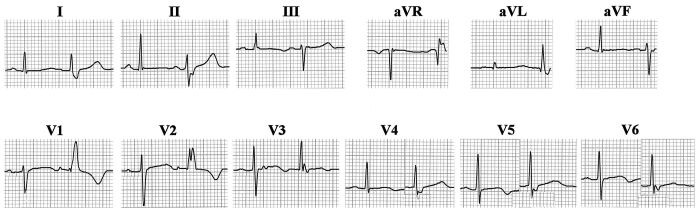
Premature atrial beats produce a consistent aberrancy of intraventricular conduction. In the two beats shown for each lead, the first is with normal conduction, and the second is aberrantly conducted. All couples of beats were consecutive except for those shown in V4 - V6, which were spliced from nonconsecutive beats. In aberrantly conducted beats, direction of middle QRS forces shifts superiorly and to the left, indicating transient LAFB, while prolonged and slurred terminal forces are directed anteriorly and to the right, indicating concomitant transient RBBB. However, loss of septal q in I indicates shift of initial forces to the left, while appearance of small q in V2 - V3 suggests also a posterior direction of initial forces, and this is most suggestive of LSFB combined with LAFB and RBBB. See text for details.

In Figure 12 is shown the ECG from a 67 year old woman with a history of hypertension and chronic chest pain. Exercise myocardial perfusion scan was normal, with normal left ventricular wall motion and ejection fraction of 68 percent. Premature atrial beats were conducted with a consistent aberrancy showing shift in initial QRS forces to the left and inferiorly; tiny q waves replaced initial r waves in leads V2 and V3. In these beats there was also left and superior deviation of the middle portion of the QRS, and rightward and anterior deviation of slowly inscribed terminal forces. The aberrant beats were consistent with LAFB combined with RBBB. However the leftward shift of initial forces and resulting loss of q wave in lead I was slightly atypical for LAFB, raising the possibility that it was combined with some degree of LSFB. Development of tiny initial q waves in V2 and V3 can result from uncomplicated LAFB, supposedly due to markedly inferior orientation of initial forces in the face of a heart that lies low in the chest relative to right precordial electrode placement [5]. Such right precordial q waves are reportedly rarely seen in LAFB in young persons [4], increasing the likelihood that, when present in the face of initial QRS forces pointing leftward, they could result from a posterior direction of initial forces [4,77], related to coexistent LSFB.

## Conclusions

That the examples illustrated above represent LSFB is predicated on the accuracy of the hypothetical models of altered ventricular activation shown in Figure 2B-2D, on which Table 1 is based. Proof that this condition is met will require additional investigation, including correlation of surface ECG patterns with electrophysiological studies of interventricular septal activation in intact humans.

Intraventricular conduction abnormalities consistent with LSFB exist and seem to be more common than has been previously recognized. As mid-late QRS forces may be minimally affected by LSFB, its diagnosis requires particular attention to changes in the direction of the initial forces of ventricular activation. Mild degrees of LSFB can mimic a normal variation [52]. Thus, comparison of ECGs taken before and after its development are usually needed for diagnosis. When the only clue to infarction limited to the interventricular septum is disappearance of septal q waves [9], it would be difficult to determine whether their loss was due to the infarction itself or to LSFB caused by the infarction.

The various electrocardiographic presentations of LSFB can tie together an assortment of otherwise inadequately explained phenomena: 1) the loss of septal q waves by leftward shift of initial QRS forces, transiently following short R-R intervals in some cases, permanently in others; 2) loss of initial r waves in right precordial leads accompanied by loss of septal q waves (which implies posterior as well as leftward orientation of initial QRS forces), either transiently or permanently as can occur with severe LVH in the absence of incomplete or compete LBBB [59,60], and in some cases of LAFB or RBBB; 3) some cases of transient right precordial Q waves during myocardial ischemia in the absence of infarction; 4) chronic QS deflections in right precordial leads in the absence of LBBB or infarction in that area; 5) atypical cases of incomplete LBBB in which left ventricular activation time remains normal and the sense of initial septal activation may not be strictly right-to-left [38,78].

This study has not addressed many important questions about the potential clinical significance of LSFB. Does the alteration of activation pattern of the interventricular septum in LSFB produce recognizable changes in its pattern of contraction? A study of this might require an analysis of septal motion more sophisticated than is currently employed in clinical echocardiography or other imaging methods [79-82]. Are there prognostic implications to the presence of LSFB? If permanent, it certainly implies the presence of disease involving at least a portion of the left bundle branch system. However, It remains for further study to show whether it is a harbinger of more advanced disease of the conduction system or myocardium, whether there are histopathologic correlations to its presence, and whether it carries prognostic significance similar to that of LAFB or LPFB [3,5].

## Figures and Tables

**Table 1:**
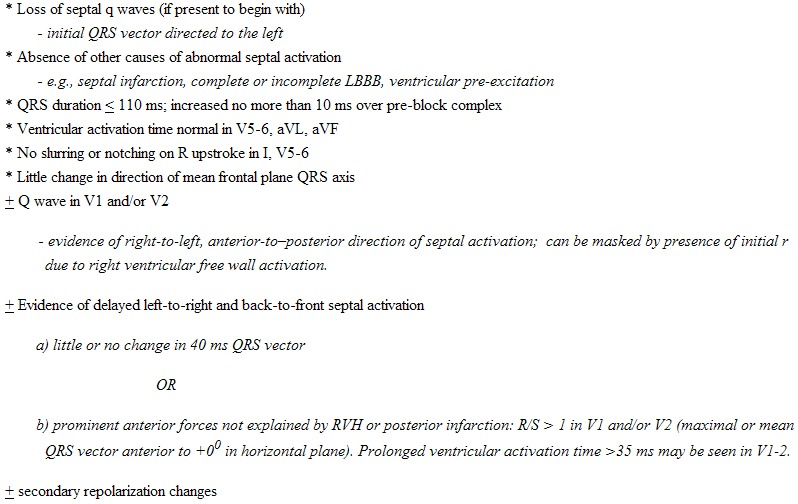
Proposed Criteria For Uncomplicated Left Septal Fascicular Block

* all items starting with an asterisk are needed for diagnosis ± indicates not necessary for diagnosis

**Table 2:**
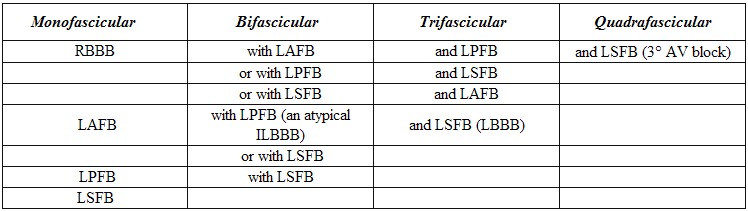
Possible Single or Multiple Fascicular Blocks in a Quadrafascicular Intraventricular Conduction System

## Abbreviations And Acronyms

ECG: electrocardiogram
ILBBB: incomplete left bundle branch block
LBBB: left bundle branch block
LAFB: left anterior fascicular block
LPFB: left posterior fascicular block
LSFB: left septal fascicular block
LVH: left ventricular hypertrophy
RBBB: right bundle branch block
